# Effects of Apple, Oak, and Mesquite Wood Chips on the Physicochemical Properties, Volatile Organic Compounds, and Sensory Characteristics of Brined Smoked Goat Meat

**DOI:** 10.3390/foods15162806

**Published:** 2026-08-11

**Authors:** Jinwoo Park, Dowon Jeong, Yousung Jung, Soomin Oh, Dongwook Kim, Aera Jang

**Affiliations:** Department of Applied Animal Science, College of Animal Life Sciences, Kangwon National University, Chuncheon 24341, Republic of Korea; jineiu5@naver.com (J.P.);

**Keywords:** goat meat, volatile organic compounds, HS-SPME-GC-MS, guaiacol, phenol, off-flavor, sensory evaluation, wood smoke

## Abstract

This study investigated the effects of apple, oak, and mesquite wood chip types on the physicochemical properties, VOC profiles, and sensory characteristics of brined smoked goat meat. Boneless muscles were brined in 1% NaCl and randomly assigned to a non-smoked control group or three wood chip-smoked groups. All smoked groups showed significantly lower TBARS values than the control (*p* < 0.05), indicating improved oxidative stability, whereas pH, cooking loss, and Warner–Bratzler shear force did not differ significantly among groups, indicating that fundamental textural properties were preserved. Notably, mesquite- and oak-smoked meats exhibited significantly higher abundances of key smoke-derived phenolic compounds—including guaiacol, phenol, o-cresol (2-methylphenol), and 2-methoxy-5-methylphenol—and sulfur-containing compounds such as carbon disulfide and hexathiane, which correlated with reduced off-flavor scores and significant improvements in smoke flavor and overall acceptability compared to the control and apple-smoked groups (*p* < 0.05). These findings demonstrate that smoking goat meat with mesquite or oak wood chips is an effective processing strategy to mask the perception of species-specific off-flavor and enhance overall sensory acceptability.

## 1. Introduction

Global consumption patterns for meat products that are both nutritious and environmentally sustainable have been steadily rising, driven by increasing consumer awareness of diet and health issues. Sheep and goat meat are widely consumed globally due to their distinctive flavor, eating quality, nutritional benefits, and cultural significance. Notably, goat meat is regarded as a relatively lean type of red meat [1]. The production of goat and sheep meat has increased markedly, primarily attributed to their favorable nutritional profiles and unique sensory properties [2]. Goat meat provides high-quality protein, B vitamins, essential fatty acids, and minerals, while containing lower fat and cholesterol levels than many conventional red meats [1,3]. However, the distinctive flavor of goat meat, arising from complex interactions among lipid components, proteins, amino acids, and volatile organic compounds (VOCs), may impede consumer acceptance due to the presence of undesirable odors [1,2,3]. Since the sensory profile of goat meat is strongly influenced by its volatile composition, which can be modulated by processing conditions and the application of smoking techniques, optimizing these strategies to regulate VOCs is essential for enhancing its eating quality and marketability [2]. The characteristic off-flavor of goat meat is primarily attributed to branched-chain fatty acids, particularly 4-methyloctanoic acid and 4-ethyloctanoic acid, which accumulate in adipose tissue through rumen metabolism [4]. Various processing strategies have been investigated to reduce this off-flavor, including marination with plant-based extracts and spices [5]; however, their effectiveness remains limited, highlighting the need for alternative approaches such as smoking.

Historically, the application of smoke has served as a primary food stabilization technique, traditionally employed to extend shelf life and protect meat from microbial spoilage [6]. However, in modern meat processing, smoking has evolved beyond preservation to serve primarily as a flavor-enhancing technique that imparts desirable sensory attributes, including color, aroma, and taste [7,8]. The generation of VOCs during smoking arises from complex interactions involving wood pyrolysis, lipid oxidation, protein degradation, and Maillard reactions [7,9]. These reactions produce diverse chemical families of volatiles, including phenolic compounds (e.g., guaiacol, 4-methylguaiacol), furans (e.g., furfural, 2-pentylfuran), aldehydes (e.g., hexanal, nonanal), ketones, and alcohols, which collectively contribute to the characteristic smoky, woody, sweet, fruity, and fatty flavor notes of smoked meat products [7,10]. Among these, phenolic compounds derived from lignin pyrolysis are considered the primary contributors to the distinctive smoky aroma [7,11]. In particular, the intense smoky, woody, and phenolic aroma notes imparted by guaiacol and cresols may competitively suppress the perception of goat-associated off-odors through olfactory masking—a phenomenon in which high-intensity odorants reduce the perceived intensity of other co-present odors [12]. This perceptual masking effect, rather than a direct chemical reaction between smoke-derived phenolics and off-flavor precursors, is proposed as the primary mechanism underlying the improvement in goat meat sensory acceptability following smoking.

The type of wood used for smoking significantly influences the volatile profile and sensory characteristics of the final product [7,11]. Wood species vary in their chemical composition, particularly in cellulose, hemicellulose, and lignin content, which directly dictates the generation of specific VOCs during thermal degradation [7,10]. Moreover, smoking parameters such as temperature, duration, smoke density, and airflow modulate the extent of wood pyrolysis and the subsequent distribution of volatile compounds [6,7,10]. Although sheep meat shares key off-flavor precursors with goat meat—including branched-chain fatty acids, skatole, and indole—studies on the volatile profiles of smoked sheep or goat meat, particularly those examining the effect of different wood chip types, remain limited in the literature, representing a critical knowledge gap in the field.

The primary objective of this study was to identify the most effective wood chip type (apple, oak, or mesquite) for modifying the VOC profile and reducing the species-specific off-flavor of smoked goat semimembranosus. Secondary objectives were to evaluate the effects of wood chip type on physicochemical properties (pH, color, cooking loss, WBSF), lipid oxidation (TBARS), and overall sensory acceptability.

## 2. Materials and Methods

### 2.1. Experimental Design

**Figure 1:**
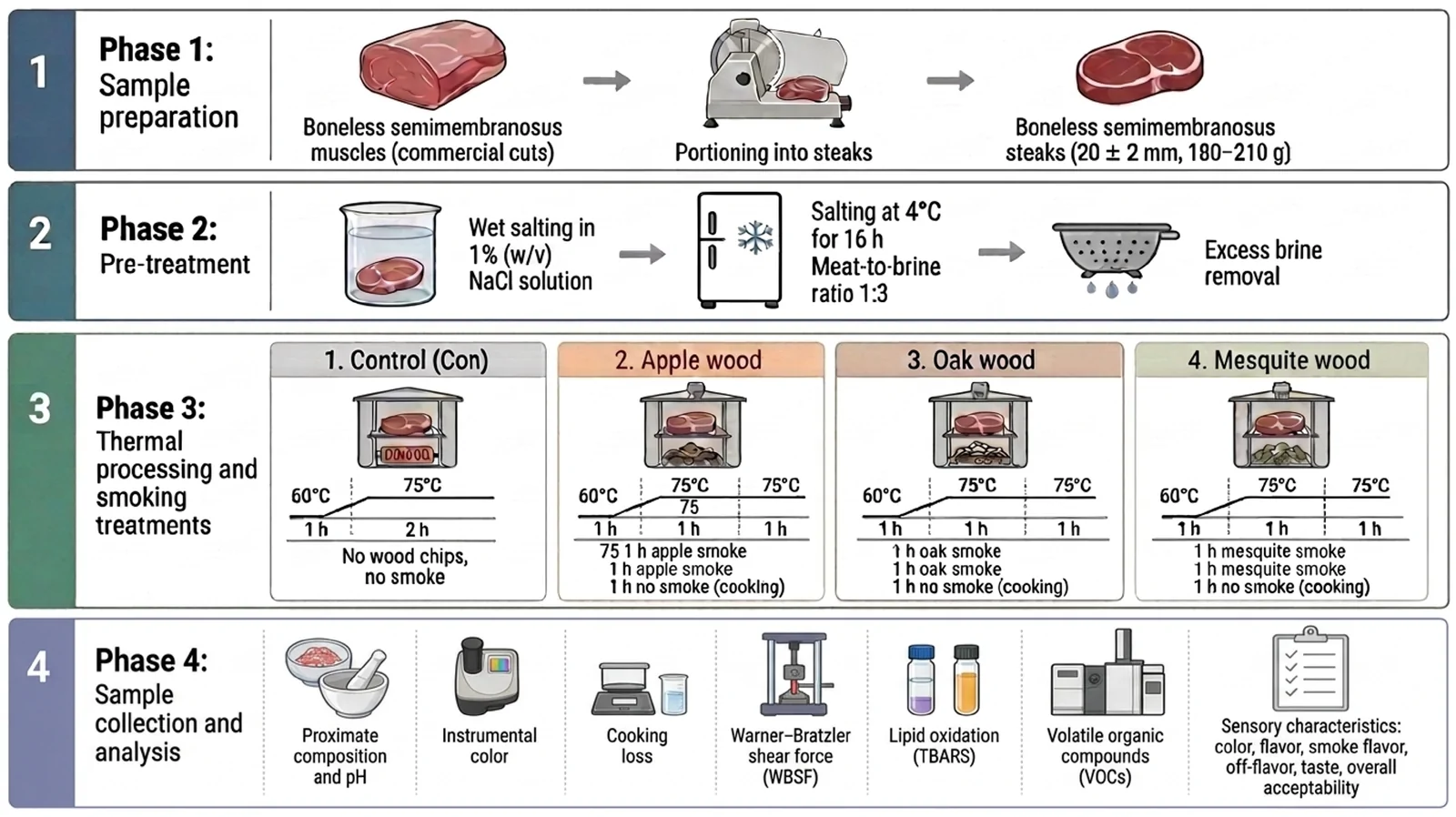
Experimental design and processing scheme for smoked goat meat treated with different wood chips. For the control group, thermal processing consisted of a continuous 1 h (60 °C) + 2 h (75 °C) sequence without interruption, distinct from the three discrete 1 h phases (60 °C smoking, 75 °C smoking, 75 °C no-smoke cooking) applied to the smoked groups. Created with Claude and finalized with Microsoft PowerPoint.

This study examined how different wood chips used for smoking influence the physicochemical properties, lipid oxidation, volatile organic compounds (VOCs), and sensory characteristics of goat meat. Boneless semimembranosus muscles were obtained as ten individually packaged frozen commercial cuts of Australian goat meat from a single supplier, of which five muscle units were used in the present experiment; the remaining units were retained as reserve stock and were not included in the analysis. From each of the five muscle units used, four steaks were cut and randomly assigned—one steak per unit to each of the four treatment groups (control, apple, oak, mesquite)—following a randomized complete block design (RCBD), with muscle unit serving as the blocking factor to control for unit-to-unit variation. This design yielded five replicates per treatment group (n = 5): a non-smoked control group and three smoked groups using apple, oak, or mesquite wood chips. Prior to thermal processing, all samples were subjected to wet salting in a 1% (*w*/*v*) NaCl solution at a meat-to-brine ratio of 1:3 (*w*/*v*) and held at 4 °C for 16 h, after which excess surface brine was removed. The control group samples were cooked in a smokehouse without smoke application at 60 °C for 1 h, followed by heating at 75 °C for an additional 2 h. In contrast, the smoked group samples were exposed to smoke generated from pre-soaked wood chips (apple, oak, or mesquite) at 60 °C for 1 h and subsequently at 75 °C for 1 h, followed by cooking without smoke at 75 °C for a further 1 h. After cooling to room temperature, samples from each group were collected for the determination of proximate composition, instrumental color, pH, cooking loss, Warner–Bratzler shear force (WBSF), thiobarbituric acid reactive substances (TBARS), VOC profiles, and sensory attributes (color, flavor, smoke flavor, off-flavor, taste, and overall acceptability), as schematically illustrated in Figure 1.

#### 2.1.1. Preparation of Goat Meat

Boneless goat semimembranosus muscles were commercially sourced, frozen immediately following slaughter, and had been stored frozen for two to five months prior to distribution. The muscles were thawed at 4 ± 1 °C under refrigerated conditions for 24 h immediately before processing.

#### 2.1.2. Salting

A standardized 1% NaCl wet brining step was applied uniformly to all groups, including the non-smoked control, to ensure equivalent baseline salt penetration, water-holding capacity, and surface moisture across all treatment groups prior to smoking, thereby allowing wood chip type to be isolated as the primary experimental variable. Thawed goat meat samples were subjected to wet salting in a 1% NaCl solution at a meat-to-brine ratio of 1:3 (*w*/*v*). Samples were fully submerged in the solution and held at 4 °C for 16 h to allow adequate salt penetration. After salting, excess surface moisture was removed before the samples were subjected to smoking.

#### 2.1.3. Smoking Materials and Procedure

Three types of food-grade wood chips were used for smoking: oak, apple, and mesquite, with a particle size of 5–10 mm. Prior to smoking, wood chips were soaked in distilled water for 30 min, after which excess surface moisture was removed. Smoking was performed using a professional smoking chamber (UKM Junior; MAUTING, Valtice, Czech Republic). Approximately 150 g of wood chips were loaded at the start of the first smoking phase (60 °C, 1 h), and an additional 150 g were added at the transition to the second smoking phase (75 °C, 1 h), for a total wood chip mass of approximately 300 g per session. For the control group, salted samples were cooked without smoking at 60 °C for 1 h, followed by continuous heating at 75 °C for an additional 2 h. For the smoked groups, salted samples were smoked at 60 °C for 1 h, then the temperature was raised to 75 °C and smoking continued for an additional 1 h, followed by cooking without smoke at 75 °C for 1 h. Between consecutive smoking sessions, the chamber intake and exhaust vents were fully opened, and the chamber door was left open for 30 min to allow complete ventilation of residual smoke and cooling of the chamber interior, thereby minimizing carryover effects between treatment groups. All four treatment groups were processed sequentially within the same smoking chamber on the same day for each replication batch, in the fixed order of control, apple, oak, and mesquite, using a freshly loaded batch of the corresponding wood chips for each smoked group. The UKM Junior chamber’s aditec MRA 515 controller regulates chamber temperature and operating time only; airflow and smoke density are not independently adjustable or quantitatively monitored. Intake/exhaust vent positions were fixed manually and kept identical across all smoked groups, a limitation for independent replication. All groups were processed using the chamber’s default settings; from an ambient start (~34 °C), the chamber reached 60 °C and 75 °C within 4 and 7 min of loading, respectively, and returned to ~32 °C after the 30 min inter-session ventilation.

### 2.2. Proximate Composition

The proximate composition of the smoked goat meat samples was analyzed following AOAC Official Method [13]. To determine moisture content, samples were dried to a constant weight at 105 °C for 12 h in a convection oven and expressed as the percentage of weight loss. Crude protein content was measured by the Kjeldahl method, crude fat was extracted with ether using a Soxhlet apparatus, and crude ash was quantified by incinerating the samples in a muffle furnace at 550 °C until complete combustion.

### 2.3. pH Value

The pH of the samples was measured according to the procedure reported in a previous study [14]. Before measurement, a pH meter (Thermo Fisher Scientific, Inc., Waltham, MA, USA) was calibrated with pH 7.0 and 4.0 buffer solutions at room temperature. The calibrated electrode was then used to record pH at five different positions within each sample.

### 2.4. Color Measurements

Color was evaluated in terms of CIE L*, a*, and b* values to characterize the surface color of the smoked goat meat. A colorimeter (CR-300; Minolta Co., Osaka, Japan) was calibrated with a standard white tile (Y = 93.60, x = 0.3134, y = 0.3194) prior to use to ensure measurement accuracy. For each sample, color was measured five times at different positions on the smoked surface, and the mean value was used for analysis.

### 2.5. Cooking Loss

Cooking loss was evaluated according to a previously described procedure [15]. Cooking loss was calculated from the difference in sample weight before and after cooking using the following equation:

(1) Cooking loss (%) = (Weight of uncooked meat − Weight of cooked meat)/Weight of uncooked meat × 100

Weight of uncooked meat refers to the sample weight recorded immediately prior to thermal processing (i.e., after salting and removal of excess surface moisture).

### 2.6. Shear Force

Shear force was measured using a texture analyzer equipped with a Warner–Bratzler shear blade. The instrument was operated at a constant test velocity (crosshead speed) of 50 mm/min [15]. Rectangular pieces (approximately 2 × 1 × 2 cm) were cut parallel to the muscle fibers, and the blade was applied perpendicular to the fiber direction. The maximum force required to cut through each sample was recorded as the Warner–Bratzler shear force value.

### 2.7. 2-Thiobarbituric Acid Reactive Substances

Lipid oxidation was quantified as 2-thiobarbituric acid reactive substances (TBARS) using a spectrophotometric assay adapted from a previously described method [16]. Smoked goat meat (5 g) was homogenized with 50 μL of 7.2% butylated hydroxyanisole (BHA) and 15 mL of distilled water for 30 s. One milliliter of the homogenate was transferred to a test tube, and 2 mL of a TBA/TCA solution (20 mM TBA/15% TCA) was added. A blank was prepared by replacing the sample with 1 mL of distilled water and adding 2 mL of the TBA/TCA solution. After incubation in a water bath at 90 °C for 15 min to allow color development, samples were chilled in cold water and centrifuged at 2000× *g* for 10 min. The absorbance of the supernatant was measured at 531 nm, and TBARS values were expressed as mg malondialdehyde (MDA)/kg of meat using the following equation:

(2) TBARS (mg MDA/kg) = (Absorbance of sample − Absorbance of blank) × 4.07

The conversion factor was empirically verified in-house using a TEP-based standard curve constructed under the exact reaction conditions of the present protocol (y = 0.0708x + 0.1558s, R^2^ = 0.9977), yielding a factor of 4.07, which was applied to all TBARS calculations reported in this study.

### 2.8. VOCs

The determination of VOCs was conducted via headspace solid-phase microextraction (HS-SPME) and gas chromatography–mass spectrometry, adopting the parameters from previous reports [17]. For VOC analysis, samples were immediately homogenized following smoking, vacuum-sealed, and stored at −18 °C until analysis. Prior to extraction, samples were thawed under refrigerated conditions (4 °C), and 5 g aliquots were transferred to sealed SPME vials and equilibrated at 60 °C for 25 min before fiber introduction. Subsequently, a DVB/CAR/PDMS fiber was introduced into the headspace for a 30 min adsorption period. The analysis was conducted utilizing an Agilent 8890 GC integrated with a 5977B MS. Separation was achieved on a DB-5MS column, with helium serving as the carrier gas at 1.3 mL/min. Regarding the oven program, the temperature was initially held at 40 °C (10 min) and gradually ramped to 250 °C at 5 °C/min. Finally, mass spectra were acquired over a scan range of 30–300 *m*/*z*. The injector temperature was set at 250 °C with splitless injection mode. The MS was operated in electron ionization mode at 70 eV, with the ion source and quadrupole temperatures maintained at 230 °C and 150 °C, respectively. Volatile compounds were tentatively identified by comparing linear retention indices (LRI), calculated from a homologous series of even-numbered n-alkanes (C8–C24), with those reported in the literature, and by matching mass spectra against the NIST 20 library (NIST/EPA/NIH Mass Spectral Library). Identification required a minimum NIST spectral match score of ≥80% and an LRI deviation within ±20 units of published reference values. VOC abundances are expressed as the sum of characteristic ion peak areas (A.U. × 10^6^). No internal standard was used; values are therefore semi-quantitative and suitable for relative, not absolute, comparison among groups.

### 2.9. Sensory Evaluation

Sensory evaluation of the smoked goat meat samples was carried out with 40 panelists recruited from the College of Animal Life Sciences at Kangwon National University, all of whom had extensive prior experience in meat sensory evaluation. While a formal training regimen or physical reference standards were not utilized, sensory calibration and alignment of scoring criteria were strictly achieved via structured group discussions and briefing sessions prior to each evaluation session. During these briefings, verbal and written guidelines were established to standardize the 9-point scale anchors among the assessors, ensuring a unified conceptual understanding of descriptive attributes such as goaty off-flavor intensity and smoke flavor intensity. For the formal assessment, cooked samples were standardized to approximately 1 × 1 × 1 cm^3^ in dimension and served immediately after being reheated in a microwave oven for 10 s under identical conditions across all treatments. All samples were coded with three-digit random numbers and presented in a randomized, balanced order to control for potential carry-over effects. Panelists evaluated the samples in individual sensory booths under uniform white lighting at room temperature (~22 °C), and room-temperature drinking water along with unsalted crackers were provided as palate cleansers between samples. Color, flavor, taste, and overall acceptability were scored from 1 (“extremely undesirable”) to 9 (“extremely desirable”), whereas off-flavor intensity and smoke flavor intensity were evaluated on a scale from 1 (“very weak”) to 9 (“very strong”). The sensory evaluation was conducted in duplicate on two separate days (n = 2 replications per panelist) to enhance data reliability. All procedures involving human participants complied with the guidelines of the Institutional Review Board of Kangwon National University (KWNUIRB-2025-04-001-001), and written informed consent was obtained from all panelists prior to participation.

### 2.10. Statistical Analysis

All data were analyzed by analysis of variance (ANOVA) using a general linear model in SAS software (version 9.4; SAS Institute, Cary, NC, USA). When a significant treatment effect was detected, Tukey’s multiple range test was applied to compare means, with statistical significance set at *p* < 0.05. Each treatment was replicated five times. As the experimental design corresponds to a randomized complete block design (RCBD) with muscle unit as the blocking factor, all models were additionally fitted with muscle unit as a block term. As this did not alter significance outcomes for any response variable, results from the unblocked model are reported hereafter for consistency with conventional reporting practice. For volatile organic compounds, data were log10-transformed and auto-scaled prior to analysis. Partial least squares-discriminant analysis (PLS-DA) was performed using the MetaboAnalyst 5.0 online platform, and group discrimination was validated by permutation testing (n = 2000). To account for multiple comparisons across 139 simultaneous ANOVA tests, the Benjamini–Hochberg false discovery rate (FDR) correction was applied to individual VOC *p*-values using the p.adjust function in R (version 4.3.1).

## 3. Results and Discussion

### 3.1. Proximate Composition

The proximate composition of goat meat samples, as influenced by different wood chip types, is detailed in Table 1.

Statistical analysis revealed no significant differences in the levels of moisture, crude protein, crude fat, and crude ash across all groups, indicating that the choice of smoking wood chips did not markedly alter the fundamental nutritional profile of the meat. Specifically, moisture contents ranged from 54.44% to 55.91%, showing only minor variations across the groups. Similarly, crude protein contents were 32.06%, 34.69%, 31.60%, and 32.72% for the control, apple, oak, and mesquite groups, respectively, while crude fat contents ranged from 8.73% to 11.24%. Crude ash contents further confirmed the absence of significant group effects, with values between 1.41% and 1.75%. This consistency aligns with previous research on smoked goat meat products, which reported no significant differences in major proximate components among treatment groups [18]. Consequently, these findings demonstrate that smoking goat meat with apple, oak, or mesquite wood chips enhances flavor while preserving the essential nutritional qualities of the product.

### 3.2. Physicochemical Properties

Table 2 presents the physicochemical properties of smoked goat meat treated with different wood chips.

The pH values of the control, apple, oak, and mesquite groups were 6.63, 6.48, 6.46, and 6.51, respectively, with no significant differences observed among groups. Fresh goat meat generally has an ultimate pH ranging from 5.5 to 6.2, which is higher than that of other red meats (5.4–5.9), because goats are more susceptible to pre-slaughter stress and have lower glycogen reserves [3]. Thermal processing is known to elevate meat pH through protein denaturation and the release of basic amino acids [19], which may explain the pH range observed across all groups in this study.

As shown in Table 2, the CIE color coordinates of the smoked goat meat varied depending on the wood chip type. Specifically, the L* values differed significantly across the groups (*p* < 0.05), with the control group exhibiting the highest lightness (32.33), followed by mesquite (28.90), apple (28.22), and oak (27.65). The CIE a* values also differed significantly (*p* < 0.05), with the control group showing the highest redness (24.84), whereas the mesquite and apple groups had lower a* values of 22.69 and 23.56, respectively, and the oak group showed an intermediate value of 24.07. Similarly, the CIE b* values showed significant differences (*p* < 0.05): The mesquite group showed the highest numerical b* value (14.86), which was not significantly different from the control group (14.34), whereas the oak group (12.97) showed significantly lower yellowness than the control group; the apple group (13.27) was intermediate and did not differ significantly from the control group (*p* < 0.05). These results indicate that smoking with different wood chips significantly modified both the lightness and color saturation of the goat meat. The reduction in lightness (L*) and redness (a*) in all smoked groups compared with the control group reflects the browning and color modification that occurs during smoke deposition on the meat surface. In contrast, the yellowness (b*) parameter showed a different pattern, with the oak group producing meat with significantly lower b* values while the mesquite group maintained higher yellowness, suggesting that wood type influences the specific smoke constituents deposited and their effects on meat pigments. Similar selective effects of smoking on meat color parameters have been reported in other meat products [20,21].

Regarding physical and textural properties, the cooking loss and Warner–Bratzler shear force (WBSF) values showed no significant differences among groups. The cooking loss values were 45.87%, 45.27%, 44.33%, and 43.81% for the control, apple, oak, and mesquite groups, respectively. Previous studies have reported that the effect of smoking on cooking loss depends more on smoking temperature, time, and method rather than wood type alone [20]. In the present study, all samples were cooked under identical conditions after smoking, suggesting that the smoking chip type itself had minimal impact on water-holding capacity [22,23]. Similarly, the WBSF values were 63.33, 56.88, 56.97, and 57.10 N for the control, apple, oak, and mesquite groups, respectively. Although the control group showed a numerically higher WBSF value than the smoked groups, the difference was not statistically meaningful. Goat meat is known to have higher shear force than other red meats [3], and while salting and mild smoking can improve tenderness [24], some studies report that high-temperature smoking increases shear force [25]. Taken together, these results imply that the relatively mild smoking conditions and the choice of apple, oak, or mesquite wood chips in the present study are unlikely to cause perceptible differences in moisture retention or tenderness for consumers.

The 2-thiobarbituric acid reactive substances (TBARS) values were 0.69, 0.54, 0.43, and 0.39 mg malondialdehyde (MDA)/kg for the non-smoked control, apple, oak, and mesquite groups, respectively (*p* < 0.05). The non-smoked control group showed significantly higher TBARS values than the apple group, which in turn was significantly higher than the oak and mesquite groups; no significant difference was observed between the oak and mesquite groups, indicating that the smoking process reduced lipid oxidation in cooked goat meat. Previous studies on various meat products have reported that conventionally smoked samples tend to show lower TBARS values than non-smoked controls, which has been attributed to the antioxidative effects of smoke-derived phenolic compounds [9]. The TBARS value observed in the non-smoked control group (0.69 mg MDA/kg) falls within the range reported for goat meat subjected to other heat-treatment methods without smoking (approximately 0.6–1.5 mg MDA/kg, depending on cooking temperature and duration) [26], while the lower values observed in the smoked groups are consistent with the additional antioxidative contribution of smoke-derived phenolic compounds described above.

### 3.3. VOCs

**Figure 2:**
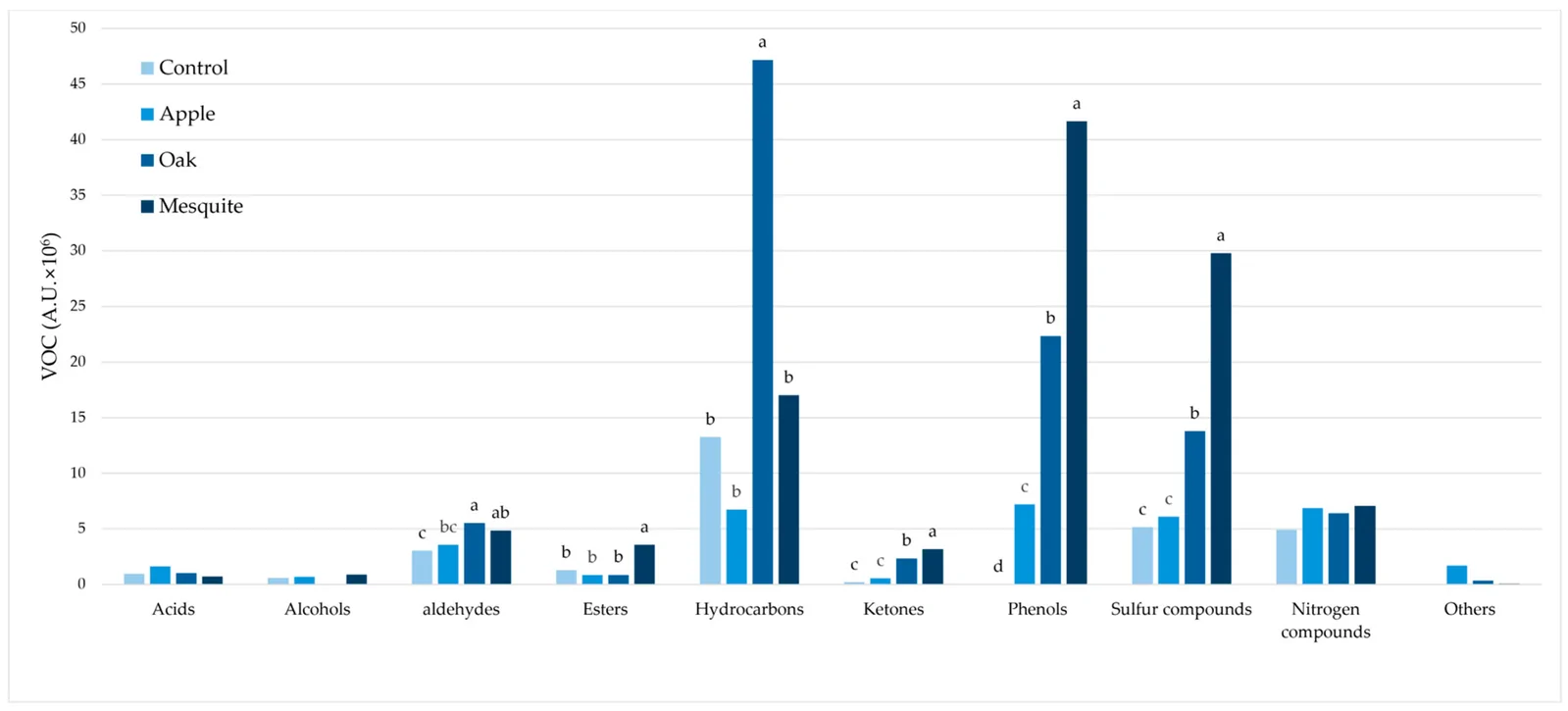
Total abundance of volatile organic compounds (VOCs) categorized by chemical class in goat meat smoked with different wood chips (A.U. × 10^6^). Different superscripts within each chemical class indicate significant differences among treatment groups (*p* < 0.05); chemical classes without superscripts indicate no significant difference among groups. Phenolic compounds constituted the most abundant VOC class in the mesquite-smoked group (41.639 A.U. × 10^6^), followed by the oak group (22.337 A.U. × 10^6^), the apple group (7.190 A.U. × 10^6^), while no phenolic compounds were detected in the control group (Supplementary Table S1). It should be noted that guaiacol, phenol, p-cresol, and o-cresol—the principal phenolic compounds discussed below—did not meet the VIP ≥  1.5 threshold and therefore do not appear in Table 3; however, these compounds exhibited markedly higher abundances in the oak and mesquite groups (Supplementary Table S1) and are discussed here in the context of their established sensory significance in smoked meat products. Among the 19 identified phenolic compounds, guaiacol (2-methoxyphenol) was the single most dominant component, with concentrations increasing from undetectable levels in the control group to 2.361, 6.197, and 15.693 A.U. × 10^6^ in the apple, oak, and mesquite groups, respectively (*p <* 0.05; Supplementary Table S1). Other major phenolics in the mesquite group included phenol (7.445 A.U. × 10^6^), 2-methoxy-5-methylphenol (6.132 A.U. × 10^6^), p-cresol (3.477 A.U. × 10^6^), and 4-ethyl-2-methoxyphenol (3.187 A.U. × 10^6^), all of which showed a consistent treatment-dependent pattern (mesquite > oak > apple > control; *p <* 0.05; Supplementary Table S1). These phenolic compounds, particularly guaiacol (smoky and sweet notes), cresols (burnt and pungent attributes), and methoxyphenols such as 2-methoxy-5-methylphenol and 4-ethyl-2-methoxyphenol (woody and spicy characteristics), are primarily responsible for the typical smoky, woody, and creosote-like flavor profile of wood-smoked meat products [12,27].

Table 3 presents the key volatile organic compounds (VOCs) with a variable importance in projection (VIP) score ≥ 1.5, identified as the primary contributors to group discrimination. The complete VOC profile is provided in Supplementary Table S1, in which a total of 139 VOCs were identified across all groups, comprising 2 acids, 4 alcohols, 11 aldehydes, 8 esters, 58 hydrocarbons, 13 ketones, 19 phenols, 4 sulfur compounds, 15 nitrogen compounds, and 5 others. One of these compounds, octamethylcyclotetrasiloxane, was identified as a probable instrumental background artifact rather than a genuine meat- or smoke-derived volatile and was therefore excluded from all VOC abundance calculations reported below. It was detected at similar levels across all treatment groups, with no significant difference among them, and was also present in the system blank, indicating an instrumental rather than treatment-related origin; because it accounted for a large share of total VOC abundance in the control and apple groups, its inclusion would have distorted comparisons among treatment groups. Excluding it did not change the overall pattern of results, and no other conclusions of this study were affected. Total VOC abundance was significantly higher in the oak and mesquite groups (99.785 and 108.765 A.U. × 10^6^, respectively) compared to the control and apple groups (29.380 and 35.842 A.U. × 10^6^, respectively) (*p* < 0.05), with no significant difference observed between control and apple groups or between oak and mesquite groups (Supplementary Table S1), indicating that wood chip type significantly altered the overall VOC profile of smoked goat meat. As illustrated in Figure 2, which depicts the total abundance of each VOC chemical class across the four groups, hydrocarbons were the most abundant chemical class in the control and oak groups (13.257 and 47.173 A.U. × 10^6^, respectively), whereas phenolic compounds were the most abundant class in the apple and mesquite groups (7.190 and 41.639 A.U. × 10^6^, respectively). The overall increase in VOC abundance in smoked groups was primarily driven by phenolic and sulfur-containing compounds, which are characteristic smoke-derived compounds that impart smoky and burnt flavors in smoked meat products [12,27]. Among all chemical classes, phenolic compounds exhibited the most pronounced treatment-dependent differences, increasing markedly in the oak (22.337 A.U. × 10^6^) and mesquite groups (41.639 A.U. × 10^6^) relative to the control (not detected) and apple groups (7.190 A.U. × 10^6^) (*p* < 0.05; Supplementary Table S1).

Sulfur compounds also showed pronounced treatment-dependent accumulation, with subtotals of 5.146, 6.086, 13.785, and 29.779 A.U. × 10^6^ in the control, apple, oak, and mesquite groups, respectively (*p <* 0.05; Supplementary Table S1), representing an approximately 5.79-fold increase in the mesquite group relative to the control. This increase was predominantly attributable to carbon disulfide (CS2), which rose from non-detectable levels in the control to 26.428 A.U. × 10^6^ in the mesquite group, accounting for approximately 89% of total sulfur compound abundance in that treatment group (Supplementary Table S1). CS2, along with hexathiane and cyclic octaatomic sulfur, collectively contributes pungent, sulphureous, and burnt notes that enhance smoky complexity [12]. The exceptionally high relative abundance of CS2 observed exclusively in the mesquite-smoked group represents a highly pronounced, reproducible species-specific volatile pattern. This is consistent with the sulfur-rich composition of mesquite wood: the wood-chip combustion-zone temperature, measured directly at 340–350 °C, exceeds the threshold for thermal decomposition of sulfur-containing precursors (~190–240 °C), and mesquite (*Prosopis* spp.) wood has been reported to contain markedly higher sulfur content (~0.5%) than typical hardwoods (0.01–0.05%) [28,29]. As CS2 was not confirmed against an authentic external standard, targeted confirmatory analysis is recommended in future work.

Aldehydes were moderately elevated in the oak and mesquite groups, with subtotals of 3.023 and 3.571 A.U. × 10^6^ in the control and apple groups and 5.516 and 4.847 A.U. × 10^6^ in the oak and mesquite groups, respectively (*p <* 0.05; Supplementary Table S1). Nonanal and octanal were the most abundant individual aldehydes, with nonanal showing significantly higher concentrations in the oak (1.971 A.U. × 10^6^) and mesquite groups (1.955 A.U. × 10^6^) compared to the control (1.150 A.U. × 10^6^) and apple groups (1.169 A.U. × 10^6^) (*p <* 0.05; Supplementary Table S1). These lipid oxidation-derived aldehydes contribute meaty, fatty, and green aroma characteristics to smoked meat products [8].

Hydrocarbons were numerically the most diverse class, comprising 58 compounds, yet showed highly variable distribution across groups. The oak group exhibited the highest hydrocarbon subtotal (47.173 A.U. × 10^6^), driven primarily by 2-methylhexane (26.271 A.U. × 10^6^) and decane (5.257 A.U. × 10^6^), whereas the control (13.257 A.U. × 10^6^), mesquite (17.023 A.U. × 10^6^), and apple groups (6.734 A.U. × 10^6^) showed substantially lower hydrocarbon abundances (Supplementary Table S1). Although hydrocarbons (including dodecane and decane) constitute the largest fraction in terms of compound number, their generally high odor thresholds (e.g., decane, ~11 mg/m^3^) [30] mean their direct sensory contribution to smoky flavor perception is considered secondary to phenolic and sulfur compounds [12]. Although naphthalene and 2-methylnaphthalene were detected at higher levels in the oak and mesquite groups (Supplementary Table S1), targeted quantification of regulated PAH4 and PAH8 compounds was not conducted in the present study; future studies should include such analyses to more comprehensively assess the food safety implications of oak and mesquite wood chip smoking.

**Figure 3:**
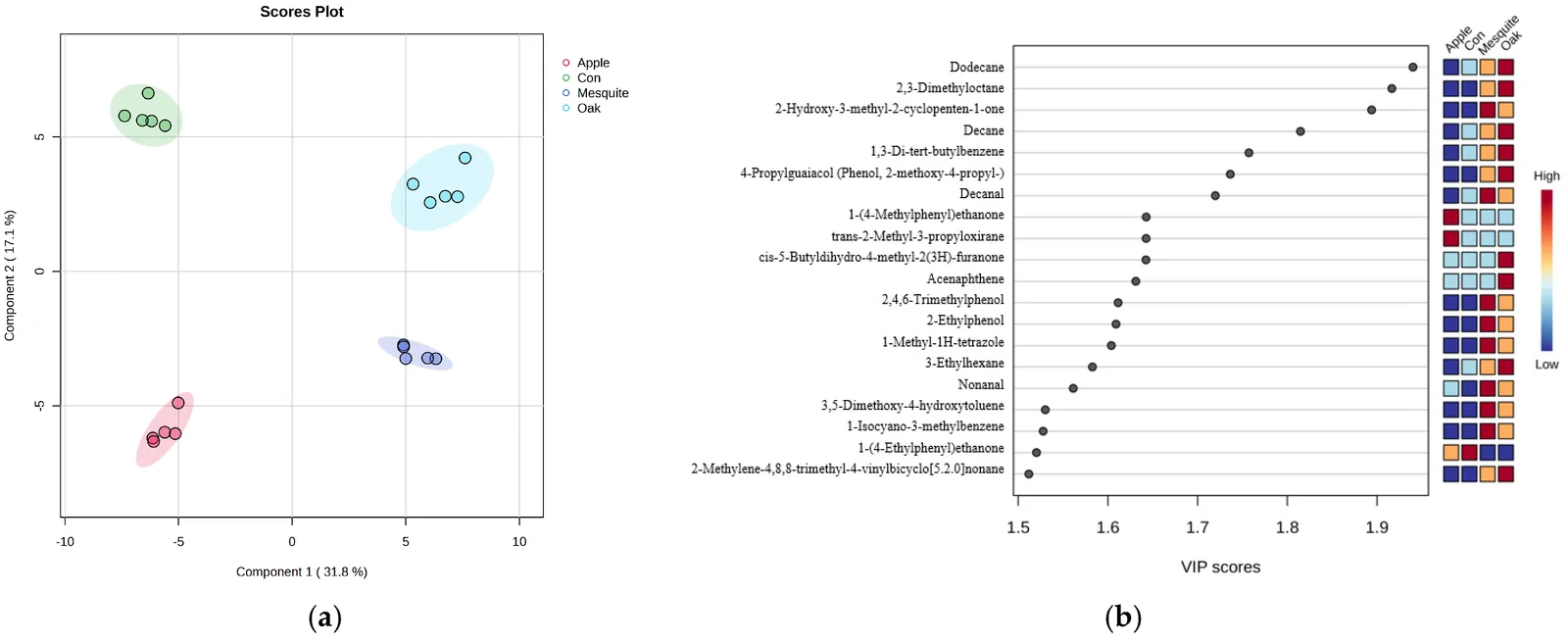
Partial least squares-discriminant analysis (PLS-DA) of volatile organic compounds (VOCs) in goat meat treated with different wood chips. (**a**) PLS-DA score plot showing clear separation among the four groups (control, apple, oak, and mesquite), indicating distinct VOC profiles according to wood type; (**b**) Variable importance in projection (VIP) plot of the top 20 VOCs (VIP ≥ 1.5) and their corresponding heatmap, illustrating the relative abundance patterns of key VOCs across the four groups (red = high, blue = low).

To further evaluate differences in VOC patterns among groups, partial least squares-discriminant analysis (PLS-DA) was conducted using all 139 identified compounds. The PLS-DA score plot clearly separated the control, apple, oak, and mesquite groups along the first two components, confirming distinct VOC profiles according to smoking treatment (Figure 3a). The model demonstrated strong explanatory and predictive performance at three components (R^2^ = 0.992, Q^2^ = 0.944), and permutation testing (n = 2000) confirmed the statistical validity of group discrimination (*p* < 0.001). Among the compounds with a VIP score ≥ 1.5, the top 20 VOCs identified as the primary contributors to group discrimination included hydrocarbons (Dodecane, Decane, 2,3-Dimethyloctane, 3-Ethylhexane), aldehydes (Decanal, Nonanal), ketones (2-Hydroxy-3-methyl-2-cyclopenten-1-one), and phenolic compounds (4-Propylguaiacol, 2-Ethylphenol, 2,4,6-Trimethylphenol) (Figure 3b, Table 3). Although guaiacol, phenol, p-cresol, and o-cresol did not rank among the top VIP contributors, these compounds exhibited markedly higher abundance in the oak and mesquite groups and are considered primary drivers of the smoky sensory characteristics observed in those treatments. It should be noted that the present analysis was based on five replicates per treatment (n = 5); while this was sufficient for detecting significant differences in physicochemical and sensory parameters, statistical power for the simultaneous analysis of 139 VOC variables is limited, and the chemometric results should be interpreted accordingly. Furthermore, brine uptake was not gravimetrically measured for individual steaks, and the three wood chip types were not characterized for moisture, ash, or lignocellulosic composition at the time of use; these factors represent potential sources of uncontrolled variation in the VOC profiles observed across treatment groups.

### 3.4. Sensory Evaluation

There were no significant differences in color scores among the groups, regardless of the wood chip type. In contrast, smoking significantly improved the sensory profile of goat meat, as indicated by higher scores for flavor, smoke flavor, taste, and overall acceptability compared to the control group (*p* < 0.05) (Table 4). For flavor, the oak and mesquite groups scored significantly higher than the control group, whereas the apple group did not differ significantly from either the control or the oak and mesquite groups. For smoke flavor, scores increased progressively from control to apple to oak to mesquite, with the mesquite group scoring significantly higher than both the control and apple groups, while the oak group did not differ significantly from either the apple or mesquite groups. Off-flavor scores were significantly reduced in all smoked groups (*p* < 0.05), suggesting that the application of wood smoke effectively masked the species-specific odors of goat meat. For overall acceptability, the oak group received the highest score, differing significantly from the control and apple groups, whereas the mesquite group did not differ significantly from either the apple or oak groups. These sensory outcomes are broadly consistent with the VOC data, in which the oak- and mesquite-smoked groups showed markedly higher levels of key smoke-derived phenolic compounds such as guaiacol, phenol, and cresols, which are known to contribute smoky and woody aroma notes and may have influenced the sensory responses observed. However, a direct correspondence between individual VOCs and specific sensory attributes cannot be established from the present data, as the sensory panel evaluated overall flavor impressions without attribute-specific compound identification; the observed associations are therefore correlative rather than causal. Among the canonical goat-specific off-flavor markers, 4-methyloctanoic acid, 4-ethyloctanoic acid, and skatole were not detected under the HS-SPME conditions employed, likely due to the limited headspace volatility of branched-chain fatty acids at 60 °C and protein binding of skatole in the cooked matrix. Indole was detected exclusively in the mesquite group at a trace level (0.029 A.U. × 10^6^; Supplementary Table S1), with no detection in the control, apple, or oak groups; this concentration is substantially lower than the levels typically associated with goat-specific off-flavor perception, and its absence in the other treatment groups suggests that its contribution to the observed sensory differences was negligible. This mesquite-specific trace is consistent with thermal pyrolysis of wood-derived tryptophan, as the measured combustion-zone temperature (340–350 °C) exceeds the ~140 °C decarboxylation threshold; this pathway is plausible specifically for mesquite, a nitrogen-fixing legume (*Prosopis* spp.) reported to be comparatively protein/nitrogen-rich, unlike the non-leguminous oak and apple used here [31,32]. Accordingly, the reduction in off-flavor intensity across all smoked groups is interpreted as a perceptual masking effect mediated by smoke-derived phenolic accumulation, rather than direct chemical reduction in off-flavor precursor concentrations.

Taken together, the sensory improvement observed in the oak- and mesquite-smoked groups appears to be primarily associated with the marked accumulation of smoke-derived compounds, particularly phenolic compounds, ketones, and sulfur-containing VOCs, rather than a measurable reduction in intrinsic meat-derived VOCs. Guaiacol, phenol, p-cresol, and 2-methoxy-5-methylphenol were more abundant in these groups, and their known smoky, woody, roasted, and phenolic aroma notes are consistent with the higher smoke flavor scores and overall acceptability, and the lower off-flavor intensity, recorded in these treatments.

Several methodological limitations should be acknowledged in interpreting these sensory findings. Odor activity values and gas chromatography–olfactometry were not determined, so the relationship between specific VOCs and perceived sensory differences remains inferential. VOC samples were homogenized immediately after smoking and stored frozen at −18 °C before analysis, whereas sensory samples were freshly reheated; although the 60 °C equilibration temperature during HS-SPME extraction approximates the serving temperature, this difference in thermal history should be considered when interpreting the instrumental–sensory relationships discussed in this section.

### 3.5. Study Limitations

Several limitations warrant consideration. Goat meat was sourced as frozen commercial cuts from a single supplier, which may limit the generalizability of these findings. The smoking chamber does not permit independent quantitative monitoring of airflow or smoke density, and treatment groups were processed in a fixed, non-randomized order within each batch; this order was nonetheless designed to bias any carryover effect conservatively rather than to inflate treatment differences. Raw meat pH prior to brining was not measured, so the relative contributions of raw material variation and thermal processing to the reported pH cannot be fully separated. Regarding VOC analysis, abundances were quantified without an internal standard and should therefore be interpreted as semi-quantitative; relatedly, carbon disulfide was not confirmed against an authentic external standard. The lag time between wood chip loading and the onset of stable smoke production was not formally recorded and varied qualitatively by wood type. As odor activity values and GC-olfactometry were not performed, and VOC and sensory samples differed slightly in thermal history prior to analysis, associations between specific VOCs and perceived sensory attributes remain inferential rather than directly established. Finally, sensory evaluation was conducted by an experienced panel rather than untrained consumers, so the results reflect descriptive sensory profiles rather than direct consumer preference. Although muscle unit was incorporated as a blocking factor at the sampling stage, reanalysis explicitly modeling this block term (Section 2.10) confirmed that all conclusions remained unaffected. Future studies addressing these points—particularly through randomized processing order, internal standard-based VOC quantification, and consumer-based hedonic testing—would further strengthen confidence in these findings.

## 4. Conclusions

This study demonstrates that smoking is an effective processing strategy to enhance the quality and sensory characteristics of goat meat without compromising its fundamental nutritional properties. While all evaluated wood chips effectively inhibited lipid oxidation and reduced the perceived intensity of species-specific off-flavors, oak and mesquite wood chips produced the most favorable outcomes. Specifically, smoking with oak or mesquite wood chips significantly increased the abundance of key smoke-derived phenolic and sulfur compounds, which contributed to the sensory masking of undesirable odors and enhanced sensory acceptability. Therefore, the application of oak or mesquite wood chips in the smoking process can be recommended as a promising approach to improve the palatability of goat meat products. These findings provide a foundational basis for broader commercial applications (see Study Limitations, Section 3.5); future replication studies incorporating large-scale consumer acceptance testing are warranted to validate the commercial viability of oak- and mesquite-smoked goat meat products.

## Figures and Tables

**Table 1:** Effect of apple, oak, and mesquite smoking chips on proximate composition of goat meat.

Trait	Control	Apple	Oak	Mesquite	SEM
Moisture	55.64	54.44	55.35	55.91	1.705
Crude protein	32.06	34.69	31.60	32.72	1.174
Crude fat	9.67	8.73	11.24	9.43	0.983
Crude ash	1.41	1.75	1.48	1.46	0.105

SEM, standard error of the mean.

**Table 2:** Physicochemical properties of goat meat smoked with different wood chips.

Trait	Control	Apple	Oak	Mesquite	SEM
pH	6.63	6.48	6.46	6.51	0.094
CIE L*	32.33 ^a^	28.22 ^bc^	27.65 ^c^	28.90 ^b^	0.289
CIE a*	24.84 ^a^	23.56 ^b^	24.07 ^ab^	22.69 ^b^	0.429
CIE b*	14.34 ^ab^	13.27 ^b^	12.97 ^c^	14.86 ^a^	0.306
Cooking loss (%)	45.87	45.27	44.33	43.81	1.568
WBSF (N)	63.33	56.88	56.97	57.10	2.796
TBARS	0.69 ^a^	0.54 ^b^	0.43 ^c^	0.39 ^c^	0.013

SEM, standard error of the mean; WBSF, Warner–Bratzler shear force; TBARS, 2-thiobarbituric acid reactive substances; ^a–c^ Means within a row with different superscripts differ significantly at *p* < 0.05.

**Table 3:** Key volatile organic compounds (VIP score ≥ 1.5) in goat meat smoked with different wood chips.

VOC (A.U. × 10^6^)	*m*/*z*	LRI	Control	Apple	Oak	Mesquite	SEM
Dodecane	57.1	1200	0.363 ^c^	0.276 ^c^	0.711 ^a^	0.537 ^b^	0.041
2,3-Dimethyloctane	69.1	963	n.d. ^c^	n.d. ^c^	0.625 ^a^	0.453 ^b^	0.0383
2-Hydroxy-3-methyl-2-cyclopenten-1-one	112	1024	n.d. ^b^	n.d. ^b^	0.653 ^a^	0.689 ^a^	0.0264
Decane	57	994	2.354 ^b^	1.334 ^b^	5.257 ^a^	3.088 ^b^	0.45
1,3-Di-tert-butylbenzene	175	1258	0.149 ^b^	0.114 ^b^	0.328 ^a^	0.342 ^a^	0.0358
4-Propylguaiacol	137	1370	n.d. ^b^	n.d. ^b^	0.204 ^b^	0.447 ^a^	0.0694
Decanal	57	1206	0.054 ^b^	0.048 ^b^	0.092 ^a^	0.101 ^a^	0.0053
3,5-Dimethoxytoluene	152	1270	n.d. ^d^	0.047 ^c^	0.205 ^a^	0.104 ^b^	0.0099
3-Ethylhexane	43	766	1.078 ^bc^	0.720 ^c^	2.219 ^a^	1.553 ^ab^	0.199
2,6-Dimethylnonane	71.1	1022	0.213 ^b^	0.127 ^b^	0.664 ^a^	0.564 ^a^	0.0761
1-(4-Methylphenyl)ethanone	119	1174	n.d. ^b^	0.030 ^a^	n.d. ^b^	n.d. ^b^	0.0006
trans-2-Methyl-3-propyloxirane	43	664	n.d. ^b^	1.650 ^a^	n.d. ^b^	n.d. ^b^	0.0791
cis-5-Butyldihydro-4-methyl-2(3H)-furanone	99.1	1327	n.d. ^b^	n.d. ^b^	0.197 ^a^	n.d. ^b^	0.0085
Acenaphthene ^†^	153	1486	n.d. ^b^	n.d. ^b^	0.011 ^a^	n.d. ^b^	0.0009
2,4,6-Trimethylphenol	121.1	1202	n.d. ^b^	n.d. ^b^	0.050 ^a^	0.069 ^a^	0.0084
2-Ethylphenol	107.1	1144	n.d. ^b^	n.d. ^b^	0.138 ^a^	0.196 ^a^	0.0218
1-Methyl-1H-tetrazole	55.1	650	n.d. ^c^	n.d. ^c^	0.468 ^b^	0.781 ^a^	0.0761
Nonanal	57.1	1110	1.150 ^b^	1.169 ^b^	1.971 ^a^	1.955 ^a^	0.1646
3,5-Dimethoxy-4-hydroxytoluene	168	1450	n.d. ^b^	n.d. ^b^	0.243 ^a^	0.209 ^a^	0.0711
1-(4-Ethylphenyl)ethanone	133	1266	0.078 ^a^	0.058 ^ab^	n.d. ^b^	n.d. ^b^	0.0148

n.d., not detected; SEM, standard error of the mean; ^a–d^ Means within a row with different superscripts differ significantly (*p* < 0.05), including groups where the compound was not detected (n.d. values substituted as zero for statistical analysis). ^†^ Compound lost statistical significance (*p* > 0.05) after Benjamini–Hochberg false discovery rate (FDR) correction for multiple comparisons (see Supplementary Table S1 for full FDR results across all 139 VOC variables).

**Table 4:** Sensory evaluation scores of goat meat processed with different wood chip smoke treatments.

Attribute	Control	Apple	Oak	Mesquite	SEM
Color	7.25	7.33	7.28	7.35	0.201
Flavor	5.10 ^b^	5.68 ^ab^	6.38 ^a^	6.43 ^a^	0.245
Off-flavor	5.18 ^a^	3.58 ^b^	2.93 ^b^	3.15 ^b^	0.303
Smoke flavor	4.13 ^c^	6.30 ^b^	7.13 ^ab^	7.38 ^a^	0.246
Taste	5.25 ^b^	6.38 ^a^	6.95 ^a^	7.00 ^a^	0.237
Overall acceptability	5.35 ^c^	6.53 ^b^	7.23 ^a^	7.05 ^ab^	0.186

Values are expressed as means (n = 40). SEM, standard error of the mean. ^a–c^ Different superscripts within the same row indicate significant differences among groups (*p* < 0.05). Color, Flavor, Taste, and Overall acceptability were scored on a 9-point hedonic scale (1 = extremely undesirable; 9 = extremely desirable). Off-flavor and Smoke flavor were scored on a 9-point intensity scale (1 = very weak; 9 = very strong).

## Data Availability

The original contributions presented in this study are included in the article/Supplementary Material. Further inquiries can be directed to the corresponding author.

## Supplementary Materials

The following supporting information can be downloaded at: https://www.mdpi.com/article/10.3390/foods15162806/s1. Table S1: Complete volatile organic compound (VOC) profiles of goat meat smoked with different wood chips (A.U. × 10^6^).

## Abbreviations

VOCs	Volatile organic compounds
WBSF	Warner–Bratzler shear force
TBARS	2-Thiobarbituric acid reactive substances
SEM	Standard error of the mean
PLS-DA	Partial least squares-discriminant analysis
VIP	Variable importance in projection
HS-SPME	Headspace solid-phase microextraction
GC-MS	Gas chromatography-mass spectrometry
ANOVA	Analysis of variance
IRB	Institutional Review Board
LRI	Linear retention index
