# Supplementary material for: Effects of Apple, Oak, and Mesquite Wood Chips on the Physicochemical Properties, Volatile Organic Compounds, and Sensory Characteristics of Brined Smoked Goat Meat
Source: Foods. 2026 Aug 11;15(16):2806. doi: 10.3390/foods15162806 (PMC13512238; doi:10.3390/foods15162806)
Supplement: Supplementary file 1 [file foods-15-02806-s001.zip › foods-4481791-supplementary.pdf]

Table S1. Complete volatile organic compound (VOC) profiles of goat meat smoked with different wood chips (A.U.×10^{6}).

VOC (A.U.×10^{6})	m/z	LRI	Control	Apple	Oak	Mesquite	SEM
Acids							
Hydrogen isocyanate	43.1	552	0.959	1.574	1.023	0.726	0.7776
Octanoic acid†	60	1176	n.d.^{b}	0.055^{a}	n.d. ^{b}	n.d. ^{b}	0.0124
Subtotal			0.959	1.629	1.023	0.726	0.7726
Alcohols							
(S)-(+)-3-Methyl-1-pentanol	56.1	785	0.2^{ab}	0.349^{a}	n.d.^{b}	n.d. ^{b}	0.0826
2-Hexyl-1-decanol	57	1787	n.d.	n.d.	n.d.	0.036	0.0094
1-Octanol	56.1	1077	0.092^{a}	0.07 ^{a}	n.d.^{b}	n.d.^{b}	0.015
1-Octen-3-ol	57	969	0.284	0.253	n.d.	0.836	0.2261
Subtotal			0.577	0.671	n.d.	0.872	0.2589
Aldehydes							
2-Methylbenzaldehyde	120	1068	n.d.^{b}	0.085^{ab}	0.184^{a}	0.081 ^{ab}	0.0374
4-Ethylbenzaldehyde	133	1178	0.194	0.194	0.181	n.d.	0.0563
4-Methylbenzaldehyde	119	1069	0.032^{a}	n.d.^{b}	n.d. ^{b}	n.d. ^{b}	0.0078
Benzeneacetaldehyde†	91.1	1043	0.077^{b}	0.168^{ab}	0.381^{a}	0.214^{ab}	0.0625
Decanal	57	1206	0.054^{b}	0.048^{b}	0.092^{a}	0.101^{a}	0.0053
Hexadecanal	57	1816	0.115	0.105	0.258	0.362	0.0722
5-Methylhexanal	43	845	0.838	1.244	1.472	1.206	0.1641
Nonanal	57.1	1110	1.150^{b}	1.169^{b}	1.971^{a}	1.955^{a}	0.1646
Octanal	43.1	998	0.533	0.463	0.882	0.828	0.1904
Pentadecanal	57	1716	0.03	0.035	0.072	0.066	0.0111
(E)-2-Nonenal†	41.1	1163	n.d.	0.059	0.022	0.035	0.0155
Subtotal			3.023^{c}	3.571^{bc}	5.516^{a}	4.847^{ab}	0.4386

Esters							
Furfuryl acetate	81.1	990	n.d.[b]	n.d. [b]	0.08[a]	n.d. [b]	0.013
Benzoyl isothiocyanate	105	935	0.296	0.859	0.759	0.832	0.158
Butyl isocyanatoacetate	57	553	n.d. [b]	n.d. [b]	n.d.[b]	2.739[a]	0.3782
Dibutyl phthalate†	149	1964	n.d.	n.d.	0.025	n.d.	0.0093
Methyl salicylate	120	1194	0.057[a]	n.d.[b]	n.d. [b]	n.d. [b]	0.0078
n-Caproic acid vinyl ester	43.1	976	0.713[a]	n.d.[b]	n.d. [b]	n.d. [b]	0.0905
Nonyl nitrate	57	1230	0.02[a]	n.d.[b]	n.d. [b]	n.d. [b]	0.0044
Propyl pyruvate†	43.1	914	0.206	n.d.	n.d.	n.d.	0.0559
Subtotal			1.293[b]	0.859[b]	0.863[b]	3.571[a]	0.4333
Hydrocarbons							
3,4-Dimethoxytoluene	152.1	1242	n.d.[c]	0.055[c]	0.229[b]	0.414[a]	0.0203
3,5-Dimethoxytoluene	152	1270	n.d. [d]	0.047[c]	0.205[a]	0.104[b]	0.0099
1,3,7-Octatrien-5-yne†	104	816	n.d.	0.59	n.d.	0.567	0.1818
2,2-Dimethyl-2,3-dihydro-1H-indene	131	1170	0.038[a]	n.d.[b]	n.d. [b]	n.d. [b]	0.007
2,4-Dimethyldodecane	43	1267	0.025[a]	n.d.[b]	n.d. [b]	n.d. [b]	0.0052
2,6,10-Trimethyltridecane	57	1464	0.054	0.066	n.d.	0.064	0.0272
2-Hexadecene,3,7,11,15-tetramethyl-,[R-[R*,R*-(E)]]-	70	1846	0.02	0.021	n.d.	n.d.	0.0065
(E)-3-Methyl-2-undecene	70.1	1187	0.035[a]	n.d.[b]	n.d. [b]	n.d. [b]	0.0051
3,7,11,15-Tetramethylhexadec-2-ene	70.1	1846	n.d.[b]	0.025[ab]	0.041[a]	0.053[a]	0.0073
Acenaphthene†	153	1486	n.d.[b]	n.d. [b]	0.011[a]	n.d. [b]	0.0009
Acenaphthylene	152	1449	n.d.[b]	n.d.[b]	0.047[a]	0.021[ab]	0.0107
Alloaromadendrene	161	1444	0.006	n.d.	n.d.	0.011	0.0029
1,3-Di-tert-butylbenzene	175	1258	0.149[b]	0.114[b]	0.328[a]	0.342[a]	0.0358
1,3-Divinylbenzene	130	1117	0.058[a]	n.d.[b]	n.d. [b]	n.d. [b]	0.0092

1-Ethenyl-4-ethylbenzene[†]	117	1088	0.494[a]	0.494 [a]	0.438 [a]	n.d.[b]	0.1173
1-Isocyano-3-methylbenzene	117	1142	n.d.[c]	n.d. [c]	0.031[b]	0.102[a]	0.0071
1-Pentenylbenzene	117.1	1136	0.041[a]	n.d.[b]	n.d. [b]	n.d. [b]	0.0095
4-Ethenyl-1,2-dimethylbenzene[†]	117	1096	n.d. [b]	0.348[a]	0.294 [a]	n.d. [b]	0.0672
4-Ethyl-1,2-dimethoxybenzene[†]	151	1328	n.d. [b]	n.d. [b]	n.d. [b]	0.04[a]	0.0083
2-Methylene-4,8,8-trimethyl-4-vinylbicyclo[5.2.0]nonane	93	1410	n.d. [b]	n.d.[b]	0.074[a]	0.041[ab]	0.0158
Decane	57	994	2.354[b]	1.334[b]	5.257[a]	3.088[b]	0.45
2,4-Dimethyldecane	43.1	1114	0.049	n.d.	0.118	0.092	0.0298
2-Methyldecane	57	1062	0.215	0.214	0.535	0.572	0.1875
3,8-Dimethyldecane[†]	57	1145	0.017	n.d.	n.d.	n.d.	0.0044
3-Methyldecane	57.1	1067	0.269	n.d.	0.549	0.638	0.2
4-Ethyldecane	43	1154	0.045[a]	n.d.[b]	n.d. [b]	n.d. [b]	0.0015
4-Methyldecane	71	1065	0.053 [ab]	n.d. [b]	0.102[ab]	0.195[a]	0.0429
Dodecane	57.1	1200	0.363[c]	0.276[c]	0.711[a]	0.537[b]	0.041
2,6,11-Trimethyldodecane	71	1284	n.d.	n.d.	0.056	n.d.	0.0141
3,3,4-Trimethylheptane	71.1	950	0.092[a]	n.d.[b]	n.d. [b]	n.d. [b]	0.0189
3,4,5-Trimethylheptane	43	941	0.195 [a]	0.04[ab]	0.212[a]	n.d.[b]	0.0456
4-Isopropylheptane	57	922	0.038[a]	n.d.[b]	n.d. [b]	n.d. [b]	0.0079
3-Ethylhexane	43	766	1.078[bc]	0.720[c]	2.219[a]	1.553[ab]	0.199
2-Methylhexane	43	662	4.082[b]	0.105[b]	26.271[a]	n.d.[b]	5.288
3-Methylhexane	43	690	n.d.	n.d.	2.134	2.87	0.8001
Naphthalene	128	1181	n.d.[d]	0.178[c]	0.939[a]	0.408[b]	0.0377
2-Methylnaphthalene	142	1312	n.d. [d]	0.015[c]	0.072[a]	0.035[b]	0.0028
2,3-Dimethylnonane	71.1	1065	0.052	n.d.	0.224	0.163	0.0634
2,5-Dimethylnonane	57	1018	0.147[b]	0.091[b]	0.429[a]	0.451[a]	0.0574

2,6-Dimethylnonane	71.1	1022	0.213^{b}	0.127^{b}	0.664^{a}	0.564^{a}	0.0761
2,3,6-Trimethyloctane	57.1	1058	n.d.	0.062	n.d.	n.d.	0.0211
2,3-Dimethyloctane	69.1	963	n.d.^{c}	n.d.^{c}	0.625^{a}	0.453^{b}	0.0383
2,4,6-Trimethyloctane	57	964	0.08	n.d.	0.194	n.d.	0.0504
2,6-Dimethyloctane†	43.1	947	n.d.^{b}	0.113^{a}	n.d.^{b}	n.d.^{b}	0.0142
3,5-Dimethyloctane	57.1	934	0.238^{a}	n.d.^{b}	n.d.^{b}	0.13^{a}	0.0301
3-Ethyloctane	57.1	956	1.195	1.048	1.849	1.428	0.2743
o-cymene	119	1021	n.d.^{b}	0.088^{a}	n.d.^{b}	n.d.^{b}	0.018
Pentadecane	57.1	1502	n.d.^{b}	n.d.^{b}	n.d.^{b}	0.181^{a}	0.0394
2,2-Dimethylpentane	43.1	633	1.032^{ab}	0.258^{b}	1.767^{a}	n.d.^{b}	0.3737
2-Methylpentane	43.1	583	n.d.^{b}	n.d.^{b}	n.d.^{b}	0.923^{a}	0.1907
1,3,3-Trimethyltricyclo[2.2.1.0(2,6)]heptane	93.1	894	n.d.	0.077	n.d.	0.451	0.1231
Tridecane	57	1284	0.051^{a}	n.d.^{b}	n.d.^{b}	n.d.^{b}	0.0061
3-Methyltridecane	57	1372	0.032^{a}	0.02^{a}	n.d.^{b}	n.d.^{b}	0.0043
Undecane	57.1	1106	0.053^{ab}	n.d.^{b}	0.069^{ab}	0.115^{a}	0.0196
2,6-Dimethylundecane	57.1	1215	0.050^{ab}	0.015^{b}	0.087^{a}	n.d.^{b}	0.0178
3-Methylundecane	57.1	1173	0.272	0.156	0.299	0.307	0.0375
5-Methylundecane	43.1	1160	0.046	0.038	0.094	0.069	0.0172
D-Limonene	68	1025	0.029	n.d.	n.d.	0.04	0.0097
Subtotal			13.257^{b}	6.734^{b}	47.173^{a}	17.023^{b}	6.5708
Ketones							
2,3-Dihydro-1H-inden-1-one	132	1282	n.d.^{c}	0.039^{b}	0.157^{a}	n.d.^{c}	0.0071
2-Methyl-1-penten-1-one	69	965	n.d.	0.034	n.d.	n.d.	0.0108
cis-5-Butyldihydro-4-methyl-2(3H)-furanone	99.1	1327	n.d.^{b}	n.d.^{b}	0.197^{a}	n.d.^{b}	0.0085
2,3,4-Trimethyl-2-cyclopenten-1-one	109	1065	n.d.^{c}	0.055^{bc}	0.111^{b}	0.317^{a}	0.0193

2,3-Dimethyl-2-cyclopenten-1-one	110	1036	n.d.^{d}	0.321^{c}	1.003^{b}	1.633^{a}	0.0736
2-Hydroxy-3-methyl-2-cyclopenten-1-one	112	1024	n.d.^{b}	n.d.^{b}	0.653^{a}	0.689^{a}	0.0264
3,4,4-Trimethyl-2-cyclopenten-1-one	124	1135	n.d.^{b}	n.d.^{b}	0.073^{ab}	0.122^{a}	0.0234
3,4-Dimethyl-2-cyclopenten-1-one†	67	989	n.d.^{b}	n.d.^{b}	0.151^{b}	0.353^{a}	0.0382
2-Nonanone	58.1	1098	0.078^{a}	n.d.^{b}	n.d.^{b}	n.d.^{b}	0.0035
3a,4,7,7a-Tetrahydro-4,7-methano-1H-indene-1,8-dione	131	1197	0.046	n.d.	n.d.	n.d.	0.0162
5-Methyl-4-hexen-3-one	55	1075	n.d.^{b}	n.d.^{b}	n.d.^{b}	0.043^{a}	0.0099
1-(4-Ethylphenyl)ethanone	133	1266	0.078^{a}	0.058^{ab}	n.d.^{b}	n.d.^{b}	0.0148
1-(4-Methylphenyl)ethanone	119	1174	n.d.^{b}	0.03^{a}	n.d.^{b}	n.d.^{b}	0.0006
Subtotal			0.201^{c}	0.537^{c}	2.346^{b}	3.158^{a}	0.1384
Phenols							
2-Methoxy-4-vinylphenol	150	1318	n.d.^{b}	n.d.^{b}	n.d.^{b}	0.195^{a}	0.0433
2-Methoxy-5-methylphenol	138.1	1192	n.d.^{c}	0.878^{bc}	3.107^{b}	6.132^{a}	0.6278
trans-Isoeugenol	164.1	1453	n.d.^{b}	n.d.^{b}	n.d.^{b}	0.087^{a}	0.0191
3,5-Dimethoxy-4-hydroxytoluene	168	1450	n.d.^{b}	n.d.^{b}	0.243^{a}	0.209^{a}	0.0711
4-Ethyl-2,6-dimethoxyphenol	167	1531	n.d.	n.d.	0.071	0.105	0.0291
Eugenol	164.1	1360	n.d.^{b}	0.035^{b}	0.153^{a}	0.159^{a}	0.0223
p-cresol	108.1	1083	n.d.^{d}	0.887^{c}	2.025^{b}	3.477^{a}	0.1334
Phenol	94.1	976	n.d.^{d}	1.785^{c}	5.622^{b}	7.445^{a}	0.3954
2,4,6-Trimethylphenol	121.1	1202	n.d.^{b}	n.d.^{b}	0.05^{a}	0.069^{a}	0.0084
2,4-Dimethylphenol	122	1153	n.d.^{b}	n.d.^{b}	n.d.^{b}	0.515^{a}	0.1089
2,6-Dimethoxyphenol	154	1354	n.d.^{b}	0.222^{ab}	0.742^{a}	0.601^{ab}	0.1529
2,6-Dimethylphenol	122	1110	n.d.^{b}	n.d.^{b}	0.139^{a}	n.d.^{b}	0.0296
2-Ethylphenol	107.1	1144	n.d.^{b}	n.d.^{b}	0.138^{a}	0.196^{a}	0.0218
2-Methoxyphenol (guaiacol)	109	1093	n.d.^{d}	2.361^{c}	6.197^{b}	15.693^{a}	0.5832

4-Propylguaiacol	137	1370	n.d.^{b}	n.d.^{b}	0.204^{b}	0.447^{a}	0.0694
2-Methylphenol (o-cresol)	108	1060	n.d.^{d}	0.480^{c}	1.679^{b}	2.092^{a}	0.0907
3,5-Dimethylphenol	107	1172	n.d.^{c}	0.095^{c}	0.450^{b}	0.682^{a}	0.0323
4-Ethylphenol	107	1170	n.d.^{b}	n.d.^{b}	n.d.^{b}	0.349^{a}	0.046
4-Ethyl-2-methoxyphenol	137	1283	n.d.^{b}	0.447^{b}	1.518^{ab}	3.187^{a}	0.4765
Subtotal			n.d.^{c}	7.190^{c}	22.337^{b}	41.639^{a}	2.0553
Sulfur compounds							
Carbon disulfide	76	545	n.d.^{c}	1.406^{c}	8.159^{b}	26.428^{a}	1.4253
Cyclic octaatomic sulfur	64	2031	0.122^{b}	0.152^{b}	0.697^{ab}	1.038^{a}	0.1776
Sulfur dioxide	64	536	4.974	4.434	4.451	2.006	1.4485
Hexathiane	64	1495	0.050^{c}	0.094^{c}	0.478^{a}	0.307^{b}	0.031
Subtotal			5.146^{c}	6.086^{c}	13.785^{b}	29.779^{a}	1.8578
Nitrogen compounds							
1-(2-Furanylmethyl)-1H-pyrrole	81	1184	n.d.^{b}	n.d.^{b}	n.d.^{b}	0.054^{a}	0.0073
1-Methyl-1H-tetrazole	55.1	650	n.d.^{c}	n.d.^{c}	0.468^{b}	0.781^{a}	0.0761
1-Propyl-1H-imidazole	110	1077	n.d.^{b}	n.d.^{b}	0.189^{a}	n.d.^{b}	0.0257
Indole	117	1297	n.d.^{b}	n.d.^{b}	n.d.^{b}	0.029^{a}	0.0062
allyl ethyl ether	57.1	570	n.d.^{b}	1.162^{a}	1.772^{a}	1.562^{a}	0.2039
2-Propen-1-amine	57	549	n.d.^{b}	n.d.^{b}	n.d.^{b}	0.27^{a}	0.0562
1-Nitroheptane	55	1151	0.204^{b}	0.340^{ab}	0.522^{a}	0.354^{ab}	0.0726
Dichloronitromethane	83	557	n.d.	n.d.	3.421	2.784	1.0839
Isocyanatomethane	57	956	n.d.	0.136	n.d.	0.518	0.1522
2,5-Dimethylpyridine	107	917	n.d.^{b}	n.d.^{b}	0.019^{ab}	0.159^{a}	0.0377
sec-Butylamine	44	584	n.d.^{b}	0.922^{a}	n.d.^{b}	n.d.^{b}	0.2029
1,4-Dimethyl-pyridinium chloride	107.1	895	n.d.^{b}	n.d.^{b}	n.d.^{b}	0.552^{a}	0.1212

Benzonitrile	103	970	0.208	0.285	n.d.	n.d.	0.0759
Diethylcyanamide	55.1	898	0.048 [a]	n.d. [b]	n.d. [b]	n.d. [b]	0.0064
Nitrous oxide	44	532	4.463	4.04	n.d.	n.d.	1.2697
Subtotal			4.923	6.885	6.391	7.063	1.7926
Others							
1,2,3-Trimethoxy-5-methylbenzene	182.1	1405	n.d. [c]	0.030[b]	0.081[a]	0.072[a]	0.0057
1,2,4,5-Tetrachloro-3,6-dimethoxybenzene	261	1724	n.d. [b]	n.d. [b]	n.d. [b]	0.016 [a]	0.0009
Octamethylcyclotetrasiloxane‡	281.1	1003	35.085	33.343	29.029	36.104	6.4087
trans-2-Methyl-3-propyloxirane	43	664	n.d. [b]	1.65 [a]	n.d. [b]	n.d. [b]	0.0791
Methacrylic anhydride	69	1044	n.d. [b]	n.d. [b]	0.27 [a]	n.d. [b]	0.0361
Subtotal			0.000	1.680	0.351	0.088	0.087
Total			29.380[b]	35.842[b]	99.785[a]	108.765[a]	6.825

n.d., not detected; SEM, standard error of the mean; [a–d] Means within a row with different superscripts differ significantly ($p < 0.05$), including comparisons involving groups where the compound was not detected (n.d. values were substituted as zero for statistical analysis). Rows without superscripts indicate no significant difference among groups ($p > 0.05$). † Compound lost statistical significance ($p > 0.05$) after Benjamini–Hochberg false discovery rate (FDR) correction for multiple comparisons across the 139 VOC variables; superscript letters for these compounds reflect uncorrected (nominal) Tukey p-values. All other superscripted compounds retained significance after FDR correction. ‡ This compound is considered a probable instrumental background artifact (m/z 281, associated with GC-MS system/septum bleed) rather than a genuine meat- or smoke-derived volatile, and was excluded from Total VOC abundance and chemical class subtotal calculations reported in Table S1, Figure 2, and Section 3.3.
